# Neoadjuvant chemotherapy enabling resection of stage IVA thymoma with myasthenia gravis and systemic lupus erythematosus: a case report

**DOI:** 10.3389/fonc.2026.1828735

**Published:** 2026-07-16

**Authors:** Carlos A. Cedeno, Edgardo Santiago, Alex R. Cedeño, Jean Lafontaine, Maria J. Marcos, Omar Concepción

**Affiliations:** 1 Hematology and Medical Oncology Section, Department of Medicine, University of Puerto Rico School of Medicine, San Juan, Puerto Rico; 2 Centro Cardiovascular de Puerto Rico y del Caribe, University of Puerto Rico School of Medicine, San Juan, Puerto Rico; 3 Department of Pathology and Laboratory Medicine, University of Puerto Rico School of Medicine, San Juan, Puerto Rico; 4 Department of Radiology, University of Puerto Rico School of Medicine, San Juan, Puerto Rico

**Keywords:** myasthenia gravis, neoadjuvant chemotherapy, paraneoplastic syndrome, systemic lupus erythematosus, thymectomy, thymoma

## Abstract

**Background:**

Thymomas are rare epithelial tumors of the anterior mediastinum associated with autoimmune disease, particularly myasthenia gravis (MG). The coexistence of thymoma, MG, and systemic lupus erythematosus (SLE) is uncommon and can complicate diagnosis due to overlapping autoimmune manifestations. Management of advanced thymoma is challenging given limited prospective data and an uncertain optimal role for neoadjuvant therapy.

**Case presentation:**

A 32-year-old woman with systemic lupus erythematosus (SLE) presented with progressive proximal muscle weakness, diplopia, and dysphagia. Examination and serologic testing confirmed MG with elevated acetylcholine receptor antibodies. Chest computed tomography showed an anterior mediastinal mass (8.7 × 6.0 × 8.8 cm) abutting major mediastinal vessels, including the left superior pulmonary vein, main pulmonary artery trunk, left atrial appendage, and left brachiocephalic vein with concern for vascular invasion, along with pleural drop metastases. CT-guided core needle biopsy demonstrated thymoma, and the disease was staged as clinical stage IVA (cT2N0M1a). She received four cycles of cisplatin, doxorubicin, and cyclophosphamide (CAP) followed by two cycles of carboplatin and paclitaxel. Neoadjuvant therapy led to significant clinical improvement, near-complete resolution of dysphagia and weakness, marked radiographic tumor regression, and disappearance of pleural lesions. She subsequently underwent median sternotomy with extended thymectomy and en bloc resection of involved mediastinal tissues. Final pathology confirmed predominantly type B3 thymoma with negative margins. Postoperative radiotherapy was administered. At latest follow-up, she had complete resolution of MG symptoms and no radiographic evidence of recurrent disease.

**Conclusion:**

This case highlights challenges in managing *de novo* stage IVA thymoma with concurrent autoimmune disease and demonstrates that neoadjuvant chemotherapy can enable complete resection of advanced disease. Unlike our prior rheumatologic report on this patient, which focused on the SLE–MG coexistence, this report emphasizes multidisciplinary oncologic management of stage IVA thymoma, including neoadjuvant chemotherapy, surgical resection, adjuvant radiotherapy, and oncologic outcomes. Detailed reporting of such cases remains important to guide treatment strategies for advanced thymoma.

## Introduction

Thymomas are rare epithelial tumors of the anterior mediastinum that may demonstrate locally aggressive behavior, including invasion of adjacent mediastinal structures and pleural dissemination (1, 2).

Thymic epithelial tumors are associated with autoimmune disorders, most commonly myasthenia gravis (MG), whereas systemic lupus erythematosus (SLE) is reported less frequently (3–7).

Complete surgical resection remains the most important prognostic factor in thymoma (8). However, patients with locally advanced or stage IVA disease with pleural dissemination may not be candidates for upfront surgery (1, 9). In such cases, neoadjuvant therapy may improve resectability, although prospective evidence guiding optimal management is limited (2, 8–10).

Here, we report a case of *de novo* stage IVA thymoma with SLE and MG, in which neoadjuvant chemotherapy resulted in substantial response and enabled complete surgical resection. The presentation has been reported separately from a rheumatologic perspective, emphasizing the coexistence of SLE, MG, and thymoma (11). In contrast, the present report focuses on the multidisciplinary oncologic management of advanced thymoma, including neoadjuvant chemotherapy, surgical resection, adjuvant radiotherapy, and outcomes. This case highlights diagnostic challenges from overlapping autoimmune syndromes and the role of multimodality therapy in achieving favorable outcomes in advanced thymoma.

## Case description

A 32-year-old woman presented with progressive fatigue, dysphagia, diplopia, and proximal muscle weakness. Dysphagia had worsened over four weeks (**Table 1**). Her past medical history included systemic lupus erythematosus (SLE) diagnosed six months earlier by a rheumatologist. She was receiving prednisone 20 mg daily, hydroxychloroquine 200 mg daily, and mycophenolate mofetil 500 mg BID. Neurologic examination showed bilateral fatigable ptosis, more pronounced on the right, diplopia, nasal speech, bulbar symptoms with dysphagia, and proximal limb weakness. Upper extremity fatigability and a positive ice pack test supported MG. The differential included SLE-related myopathy, steroid-induced myopathy, and inflammatory myositis; however, ocular and bulbar involvement with fatigable weakness favored MG.

**Table 1:** Clinical timeline and key diagnostic findings.

Date	Clinical event	Key findings/treatment
03/2024	Initial presentation	Dysphagia, diplopia, ptosis, generalized weakness; ICU admission for myasthenic crisis
03/2024	Imaging and diagnosis	Anterior mediastinal mass with pleural nodules; biopsy confirmed WHO type AB thymoma
03–04/2024	Autoimmune evaluation	MG diagnosed in the setting of SLE; treated with IVIG, corticosteroids, and pyridostigmine
04–10/2024	Neoadjuvant chemotherapy	CAP ×4 → Carboplatin/Paclitaxel ×2; partial response and improved resectability
02/2025	Surgical resection	Extended thymectomy; final pathology: ypT2N0M1a, predominantly type B3 thymoma
05/2025	Adjuvant radiotherapy	Postoperative mediastinal radiotherapy completed
08/2025-03/2026	Follow-up	Resolution of MG symptoms; stable SLE with mild arthralgias; residual grade 1 sensory neuropathy; no radiographic recurrence

## Diagnostic assessment

### Initial laboratory evaluation

Initial laboratory studies were consistent with active SLE, with positive antinuclear antibodies (ANA 1:160), elevated anti-double-stranded DNA antibodies (1:160 and 33 IU/mL), hypocomplementemia (C3–71 mg/dL and C4 6.6 mg/dL), and an erythrocyte sedimentation rate of 25 mm/hr. Additional autoantibodies, including antiphospholipid antibodies, ANCA, anti-Smith/RNP, and Scl-70, were negative. Given bulbar symptoms and suspected neuromuscular junction involvement, serologic testing for MG was performed and demonstrated markedly elevated acetylcholine receptor antibodies, with binding antibodies at 122.75 nmol/L, blocking antibodies at 39% inhibition, and modulating antibodies at 95% inhibition, confirming the diagnosis.

### Imaging evaluation

Computed tomography of the chest with contrast demonstrated a lobulated solid enhancing mass in the anterior mediastinum measuring approximately 8.7 × 6.0 × 8.8 cm. The mass contained punctate calcifications and extended toward the posterior mediastinum, abutting the left superior pulmonary vein, pulmonary artery trunk, left atrial appendage, and left brachiocephalic vein with abnormal contour concerning for vascular invasion. Anteriorly, the lesion abutted the chest wall. Multiple pleural nodules consistent with pleural drop metastases were also identified. A small pericardial effusion was present (**Figure 1**). Imaging of the abdomen, pelvis, brain, and PET-CT showed no extrathoracic metastatic disease. A baseline chest MRI was obtained before systemic therapy to characterize mediastinal disease and its relationship to cardiovascular structures.

**Figure 1:**
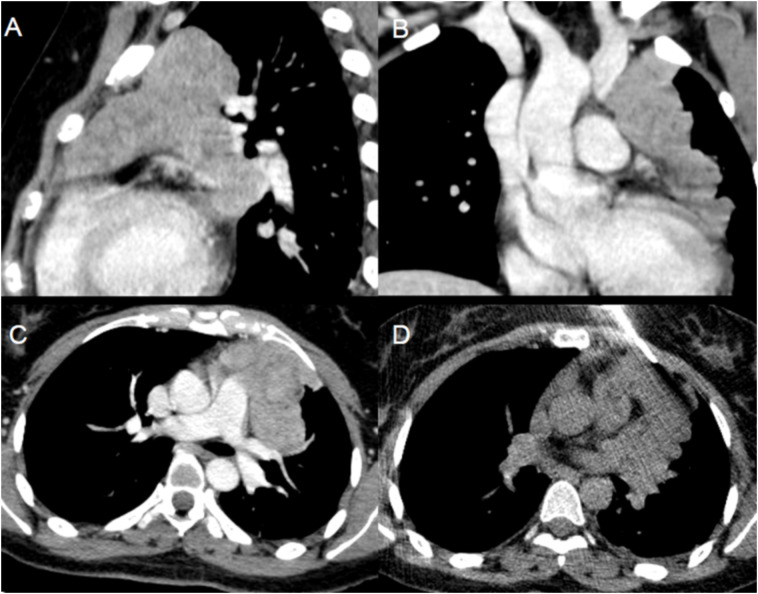
Contrast-enhanced chest CT demonstrating a lobulated solid mass measuring 8.7 × 6.0 × 8.8 cm in the left paramedian anterior mediastinum extending toward the posterior mediastinum and abutting the main pulmonary artery, left superior pulmonary vein, and left atrial appendage. The lesion produces mass effect on the left brachiocephalic vein with suspected vascular invasion. **(A)** sagittal, **(B)** coronal, and **(C)** axial contrast-enhanced images. **(D)** axial non-contrast CT obtained during core needle biopsy. Adapted with permission from González-Ramos et al., Case Reports in Rheumatology, doi: 10.1155/crrh/7670778.

### Pathologic diagnosis

Diagnosis was established by CT-guided core needle biopsy of the anterior mediastinal mass. Histopathologic examination showed thymoma type AB by World Health Organization (WHO) classification. The tumor was composed of spindle-shaped epithelial cells with bland oval nuclei and scattered immature lymphocytes. Immunohistochemistry showed epithelial cells positive for cytokeratins, p40, p63, PAX8, and BCL-2 with low Ki-67, and lymphocytes positive for CD3 (**Figure 2**).

**Figure 2:**
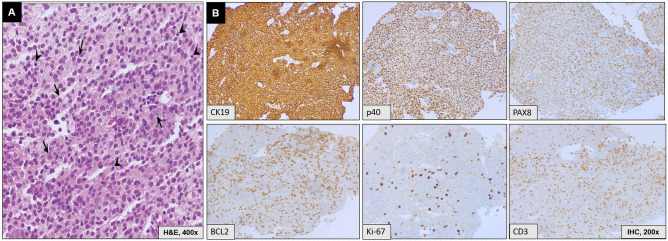
Histopathology and immunohistochemistry of the mediastinal tumor. **(A)** CT-guided core biopsy of the thymic mass. H&E stain (400×) showing bland spindle-to-oval thymic epithelial cells (arrows) admixed with small lymphocytes (arrowheads). **(B)** Immunohistochemistry (200×) demonstrating CK19, p40, PAX8, and BCL2 positivity in thymic epithelial cells, with a low Ki-67 proliferative index and admixed CD3-positive T lymphocytes.

### Staging

Given pleural dissemination and invasion of mediastinal structures, the tumor was staged as clinical stage IVA thymoma (cT2N0M1a) according to the AJCC 8th edition TNM system, corresponding to modified Masaoka-Koga stage IVA.

## Therapeutic intervention

### Clinical course and initial management

Due to bulbar symptoms and concern for impending respiratory failure, the patient was admitted to the intensive care unit for suspected myasthenic crisis. She was treated with intravenous immunoglobulin (0.4 g/kg/day for 5 days) in addition to pyridostigmine and corticosteroids, resulting in rapid improvement with near resolution of dysphagia and extremity weakness within one week. Hydroxychloroquine was discontinued because of concern for worsening myasthenic symptoms. Her immunosuppressive therapy for SLE, including mycophenolate mofetil and prednisone, was subsequently resumed.

### Multidisciplinary evaluation and neoadjuvant therapy

The case was discussed at a multidisciplinary tumor board with thoracic surgery, medical oncology, and radiation oncology. Given locally advanced disease with pleural involvement and borderline resectability, the team proceeded with neoadjuvant chemotherapy to improve resectability. The patient received the CAP regimen consisting of cisplatin 50 mg/m², doxorubicin 50 mg/m², and cyclophosphamide 500 mg/m² intravenously on day 1 every 3 weeks. She completed four cycles with sustained clinical improvement, including near-complete resolution of dysphagia and extremity weakness after the first cycle. Treatment was generally well tolerated, with only mild grade 1 peripheral sensory neuropathy. Given the objective of maximizing tumor response and improving the likelihood of complete (R0) resection, the multidisciplinary team selected the CAP regimen, a platinum-anthracycline combination commonly used as first-line therapy for advanced thymoma because of its established activity and response rates.

### Response to neoadjuvant therapy

For preoperative restaging and surgical planning, chest MRI was obtained to evaluate residual tumor extent and its relationship to mediastinal and cardiovascular structures following neoadjuvant therapy. Follow-up chest MRI performed after completion of four cycles demonstrated a partial radiographic response, with reduction of the anterior mediastinal mass from 8.7 × 6.0 × 8.8 cm at baseline to approximately 7.3 × 6.0 × 4.0 cm. Previously identified pleural nodules were no longer visualized, consistent with radiographic resolution of pleural dissemination. Despite this response, the residual mediastinal mass continued to abut the distal right ventricular outflow tract, raising concern for persistent involvement. Given the persistent close relationship of the residual tumor to cardiovascular structures, particularly the right ventricular outflow tract, and the goal of maximizing the likelihood of complete (R0) resection, the thoracic surgery team reevaluated the case and recommended two additional cycles of systemic chemotherapy before definitive surgical management. The patient subsequently received carboplatin AUC 6 intravenously on day 1 plus paclitaxel 200 mg/m² intravenously on day 1 every 3 weeks, completing a total of six cycles of neoadjuvant therapy. The regimen was changed to carboplatin and paclitaxel to allow further cytoreduction while limiting cumulative anthracycline exposure and cardiotoxicity.

### Preoperative imaging

A repeat contrast-enhanced chest MRI obtained preoperatively demonstrated interval reduction in tumor burden. The anterior mediastinal mass measured approximately 6.3 × 6.0 × 4.1 cm, representing continued response compared with prior imaging. The mass remained lobulated and enhancing and continued to abut the right ventricular outflow tract without an intervening fat plane. Previously described pleural metastases remained nearly imperceptible, with no new pleural disease or malignant effusion. No pericardial effusion or additional mediastinal or parenchymal abnormalities were noted. Overall findings were consistent with a partial radiographic response to neoadjuvant chemotherapy (**Figure 3**).

**Figure 3:**
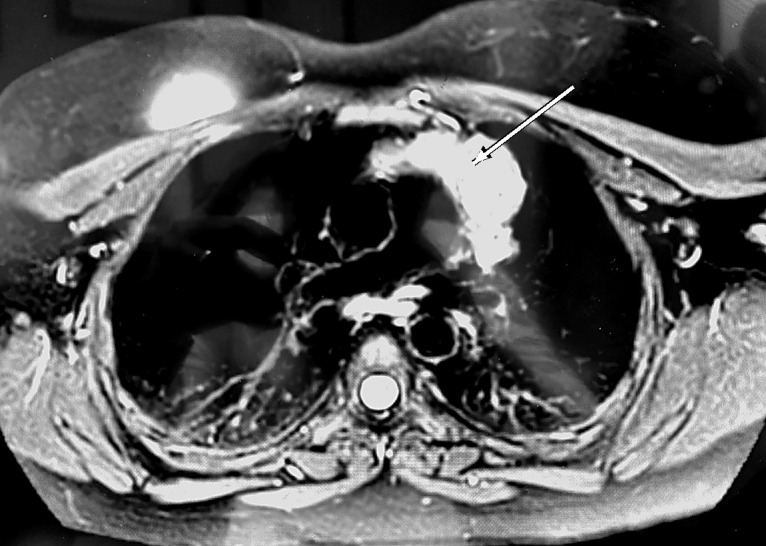
Contrast-enhanced axial magnetic resonance imaging (MRI) of the chest demonstrating a lobulated enhancing mass in the left anterior mediastinum (white arrow), abutting the right ventricular outflow tract without a preserved intervening fat plane. Compared with the previous MRI, the mass demonstrates a further decrease in size, measuring 6.3 × 6.0 × 4.1 cm compared with 7.3 × 6.0 × 4.0 cm, consistent with a continued partial radiographic response to neoadjuvant chemotherapy and improved surgical resectability.

### Surgical management

Following completion of neoadjuvant chemotherapy and radiographic evidence of tumor response, the patient underwent surgical resection. Median sternotomy was selected because preoperative imaging continued to demonstrate close tumor contact with cardiovascular structures, raising concern for locally advanced disease and the potential need for vascular or extended *en bloc* resection to achieve complete oncologic clearance. A median sternotomy with extended thymectomy was performed. Intraoperative findings revealed tumor adherence to the innominate vein requiring partial venous resection with primary venorrhaphy. The tumor also demonstrated invasion of the pericardium and encasement of the left phrenic nerve, necessitating *en bloc* resection and pericardial reconstruction. Tumor adherence to the left upper lobe required pneumorrhaphy following tumor detachment. Both pleural cavities were systematically explored, with no residual macroscopic pleural implants identified. These findings raised concern for locally advanced disease beyond preoperative imaging. Under AJCC 8th edition criteria, definitive invasion of either the innominate vein or phrenic nerve would be consistent with T3 disease, but final pathologic staging was based on surgical pathology findings documenting invasion of the mediastinal and pericardial pleura without definitive histopathologic confirmation of venous wall or phrenic nerve invasion.

## Follow-up and outcomes

### Pathologic findings

Final surgical pathology demonstrated type B3 thymoma (97%) with a minor type B2 component (3%) according to WHO classification. The tumor measured 5.2 × 4.0 × 2.5 cm and demonstrated lymphovascular invasion with approximately 70% residual viable tumor. Direct invasion into the mediastinal and pericardial pleura was identified. Surgical margins were negative, with the closest margin measuring less than 1 mm. No regional lymph nodes were identified. Immunohistochemistry showed epithelial cells positive for AE1/AE3, p63, p40, and PAX8 and scattered immature T lymphocytes positive for CD3, CD5, CD1a, TdT, and CD99. Final pathologic staging was ypT2N0M1a according to the AJCC 8th edition TNM system, corresponding to modified Masaoka-Koga stage IVA disease. The ypT2 designation was based on documented invasion of the mediastinal and pericardial pleura. Although intraoperative findings raised concern for innominate vein involvement and phrenic nerve encasement requiring resection, definitive histopathologic involvement of these structures was not reported in the final pathology specimen.

### Postoperative course

The patient had a favorable postoperative recovery, notable for mild hoarseness attributed to recurrent laryngeal nerve injury and asymptomatic elevation of the left hemidiaphragm secondary to phrenic nerve resection. She subsequently received adjuvant radiotherapy; however, detailed treatment parameters were unavailable. Following multimodality treatment, the patient experienced complete resolution of MG symptoms, including visual symptoms, ptosis, generalized weakness, and dysphagia. Her SLE remained clinically stable, with persistent arthralgias but no additional manifestations. Follow-up chest CT obtained approximately six months after surgery demonstrated expected postoperative changes without radiographic evidence of recurrent disease. At the most recent follow-up, approximately two years after initial diagnosis and 13 months after surgical resection, there was no radiographic evidence of recurrent disease.

## Discussion

This case highlights important therapeutic considerations in the multidisciplinary management of advanced thymoma, including challenges from concurrent autoimmune disease and the role of multimodality therapy in achieving complete resection.

Thymomas are associated with autoimmune syndromes, most commonly myasthenia gravis (MG), whereas systemic lupus erythematosus (SLE) is less frequent. The coexistence of thymoma, MG, and SLE can complicate diagnosis when symptoms like dysphagia and weakness overlap with autoimmune manifestations and delay recognition of MG; in this case, symptoms were present for several months before diagnosis (3–7).

Thymectomy can improve autoimmune manifestations, particularly MG. In this case, the patient experienced marked improvement in MG and SLE after multimodality treatment (3, 11).

This case highlights the multidisciplinary management of advanced-stage thymoma. Multimodality treatment with systemic therapy, surgery, and postoperative radiotherapy is often required for advanced disease or high-risk pathologic features. Retrospective series in stage III and IVA thymoma suggest that neoadjuvant chemotherapy may improve resectability and long-term survival. In one series using induction chemotherapy, surgery, and postoperative radiotherapy, multimodality treatment achieved 10-year survival of 45% to 48% (12). Database analyses also show improved survival with surgical resection combined with radiotherapy, with 5-year overall survival of about 70% for stage III and 58% for stage IV disease (9). However, optimal sequencing remains uncertain given limited prospective data, and current recommendations rely on retrospective institutional experience (2, 13).

In the present case, neoadjuvant chemotherapy with CAP followed by carboplatin and paclitaxel led to marked clinical and radiographic improvement, with near resolution of myasthenic symptoms, marked tumor reduction, and disappearance of pleural metastases on imaging. This response allowed reevaluation and conversion of a borderline resectable tumor to resectable disease (12, 13).

The selection and sequencing of neoadjuvant therapy were guided by the objective of maximizing tumor response and achieving complete (R0) resection. CAP (cisplatin, doxorubicin, and cyclophosphamide) was chosen as initial therapy because platinum-anthracycline combinations have demonstrated the highest response rates in advanced thymoma and remain widely used in the neoadjuvant setting. In the ECOG/SWOG/SECSG intergroup study by Loehrer et al., CAP achieved an overall response rate of 50% with a median overall survival of 37.7 months in patients with advanced thymoma (14). More recently, Ma et al. reported superior 5-year overall survival among patients with stage III/IVA thymoma treated with CAP compared with cisplatin/etoposide or cisplatin/paclitaxel (84.9% vs. 70.6% vs. 60.0%, respectively) (15). Despite a favorable initial response after four cycles, restaging MRI continued to demonstrate close contact between the residual tumor and adjacent cardiovascular structures, particularly the right ventricular outflow tract, raising concern regarding the feasibility of complete resection. Following multidisciplinary reassessment, two additional cycles of chemotherapy were administered to maximize cytoreduction before surgery. Carboplatin and paclitaxel were selected rather than continued CAP to limit cumulative anthracycline exposure while maintaining antitumor activity, supported by prospective data demonstrating objective responses in advanced thymoma (16). This strategy resulted in further tumor regression and ultimately facilitated complete surgical resection. In this case, MRI proved particularly valuable for surgical planning because of its superior soft-tissue characterization and assessment of relationships between the residual tumor and adjacent cardiovascular structures.

Surgical resection remains the cornerstone of treatment for advanced thymoma and a major determinant of long-term survival (8, 13). Complete (R0) resection is the most important prognostic factor and should remain the primary objective whenever surgery is pursued. A recent meta-analysis demonstrated that incomplete resection was the strongest predictor of poor overall survival (HR 4.41, 95% CI 3.32–5.85), exceeding the prognostic impact of advanced stage or histologic subtype (17). In the present case, *de novo* stage IVA thymoma with pleural dissemination responded sufficiently to neoadjuvant therapy to permit complete resection with negative margins, highlighting the potential value of a multidisciplinary multimodality approach in selected patients with advanced disease.

Under the AJCC 8th edition TNM classification, invasion of the innominate vein or phrenic nerve is categorized as T3 disease (18). In the present case, partial innominate vein resection and en bloc phrenic nerve resection were required to achieve complete resection, reflecting the locally advanced nature of the tumor. However, the final pathology report documented invasion of the mediastinal and pericardial pleura without definitive histopathologic confirmation of venous wall or phrenic nerve invasion. Accordingly, the final pathologic stage was retained as ypT2N0M1a based on the documented pathologic findings. Regardless of the T-category uncertainty, the presence of pleural dissemination remained consistent with modified Masaoka-Koga stage IVA disease (19).

The surgical approach was influenced by persistent concern for invasion of mediastinal structures despite a favorable response to neoadjuvant therapy. Restaging imaging continued to demonstrate close contact between the residual tumor and adjacent cardiovascular structures without preserved tissue planes, prompting selection of median sternotomy to facilitate complete resection and permit vascular or extended *en bloc* resection if required. Intraoperative findings ultimately confirmed greater local invasion than could be definitively established radiographically, including tumor adherence to the innominate vein and encasement of the left phrenic nerve. Although minimally invasive thymectomy has demonstrated outcomes comparable to open approaches in selected patients with stage I–III thymoma, these data do not adequately address stage IVA disease requiring potential multistructure resection or vascular reconstruction (20). Following neoadjuvant therapy, radiographic pleural lesions resolved and systematic intraoperative pleural exploration revealed no residual macroscopic disease; therefore, more extensive pleural procedures such as pleurectomy/decortication or extrapleural pneumonectomy were not pursued. Prior studies suggest that resection of the primary tumor may still provide meaningful locoregional disease control and progression-free survival benefit in selected patients with stage IVA thymoma (10).

There was a discrepancy between the initial core needle biopsy and the final surgical specimen. The biopsy showed type AB thymoma, whereas final pathology revealed predominantly type B3 thymoma with a minor B2 component, consistent with the heterogeneity of thymomas and sampling limitations of core biopsy (2, 21, 22). Although corticosteroids can induce regression in some thymomas, prolonged prednisone exposure was unlikely to explain the marked radiographic response, given the predominant B3 histology on resection; however, corticosteroids may have altered histology by reducing the lymphocytic component and unmasking the epithelial-predominant architecture (23). Histologic upgrading is well described and can affect prognostic stratification and treatment (21), and B3 thymomas are associated with more aggressive behavior than type A or AB thymomas (24). High-risk features included lymphovascular invasion, pleural involvement, and a margin of less than 1 mm, supporting adjuvant radiotherapy per NCCN guidelines. Although no discrete lymph nodes were identified on final pathology, an *en bloc* mediastinal resection was done after neoadjuvant therapy.

This report has several limitations. Long-term follow-up is short for an indolent tumor, and radiotherapy details were not available. Electromyography was not performed because it was not available. Serial postoperative autoantibody titers were not obtained, limiting assessment of immunologic treatment effects. Nevertheless, this case illustrates the role of multimodality treatment in achieving favorable oncologic and clinical outcomes in advanced thymoma with rare autoimmune manifestations.

Given the rarity of thymoma and the limited prospective evidence guiding treatment decisions, reporting of individual cases is important to inform future strategies (2, 9). The initial autoimmune presentation of this patient has been described separately from a rheumatologic perspective (11), whereas the present report focuses on the multidisciplinary oncologic management of *de novo* stage IVA thymoma, detailing a multimodality strategy with neoadjuvant chemotherapy, surgery, and adjuvant radiotherapy that enabled complete resection and durable disease control. The principal contribution of this report is the description of a multimodality treatment strategy that enabled complete resection of advanced disease and durable disease control.

## Conclusion

This case highlights the challenges of managing *de novo* stage IVA thymoma with concurrent autoimmune disease and demonstrates the value of a multidisciplinary approach. Neoadjuvant chemotherapy led to marked tumor regression, resolution of pleural lesions, and enabled complete resection despite stage IVA disease. Unlike a prior rheumatologic report on this patient, which focused on the coexistence of systemic lupus erythematosus and myasthenia gravis, the present report emphasizes multidisciplinary oncologic management of advanced thymoma with neoadjuvant therapy, surgery, adjuvant radiotherapy, and clinical outcomes. Given the rarity of advanced thymic epithelial tumors and limited prospective evidence, reports of multimodality treatment remain important to inform practice, particularly in stage IVA disease, where neoadjuvant therapy may facilitate complete resection.

### Key learning points

• Advanced stage IVA thymoma with pleural dissemination may remain amenable to curative-intent multimodality treatment in selected patients.
• Neoadjuvant chemotherapy can facilitate complete (R0) resection by reducing tumor burden and improving surgical resectability in locally advanced thymoma.
• Histologic classification based on small preoperative biopsy specimens may underestimate tumor aggressiveness because of sampling limitations and intratumoral heterogeneity, potentially affecting prognostic assessment and treatment planning.

### Patient perspective

Following treatment, the patient experienced significant improvement in her quality of life, including resolution of dysphagia, diplopia, generalized weakness, and other myasthenic symptoms that had substantially limited her daily activities before diagnosis. She was able to return to work as a cook and resume her usual routine with only mild residual peripheral sensory neuropathy and intermittent arthralgias. The patient expressed gratitude toward the multidisciplinary oncology and surgical teams involved in her care and satisfaction with the overall treatment outcome.

## Data Availability

The original contributions presented in the study are included in the article/supplementary material. Further inquiries can be directed to the corresponding author.
